# Expression of a Chromoplast-Specific Lycopene β-Cyclase Gene (*CYC*-*B*) Is Implicated in Carotenoid Accumulation and Coloration in the Loquat

**DOI:** 10.3390/biom9120874

**Published:** 2019-12-13

**Authors:** Min Hong, Zhuo-Heng Chi, Yong-Qing Wang, Yue-Ming Tang, Qun-Xian Deng, Ming-Yang He, Ri-Kui Wang, Yi-Zhong He

**Affiliations:** 1 Citrus Research Institute, Southwest University/Chinese Academy of Agricultural Sciences, Chongqing 400712, China; cnyyxyhm@163.com (M.H.); hemingyang@cric.cn (M.-Y.H.); wangrikui@cric.cn (R.-K.W.); heyizhong@cric.cn (Y.-Z.H.); 2 College of Horticulture, Sichuan Agricultural University, Chengdu 611130, China; chizhuoheng0331@gmail.com (Z.-H.C.); dqxlwj@sina.com (Q.-X.D.); 3 Institute of Agro-products Processing Science and Technology, Sichuan Academy of Agricultural Sciences, Chengdu 610066, China; tangyueming@stu.sicau.edu.cn

**Keywords:** loquat, carotenoids, *CYC*-*B* gene, virus-induced gene silencing (VIGS)

## Abstract

Carotenoids are the principal pigments in the loquat. Although the metabolic pathway of plant carotenoids has been extensively investigated, few studies have been explored the regulatory mechanisms of loquat carotenoids because knowledge of the loquat genome is incomplete. The chromoplast-specific lycopene β-cyclase gene (*CYC*-*B*) could catalyze cyclization of lycopene to β-carotene. In this study, the differential accumulation patterns of loquat with different colors were analyzed and virus-induced gene silencing (VIGS) was utilized in order to verify *CYC*-*B* gene function. Using a cloning strategy of homologous genes, a *CYC*-*B* gene orthologue was successfully identified from the loquat. At a later stage of maturation, *CYC*-*B* gene expression and carotenoids concentrations in the ‘Dawuxing’ variety were higher than in ‘Chuannong 1-5-9′, possibly leading to the difference in pulp coloration of loquat. Interference of *CYC*-*B* gene expression in the loquat demonstrated clear visual changes. The green color in negative control fruits became yellow, while *TRV2-CYC-B* silenced fruits remained green. *CYC*-*B* gene expression and total carotenoid content in the pulp decreased by 32.5% and 44.1%, respectively. Furthermore, multiple key genes in the carotenoid metabolic pathway synergistically responded to downregulation of *CYC*-*B* gene expression. In summary, we provide direct evidences that *CYC*-*B* gene is involved in carotenoid accumulation and coloration in the loquat.

## 1. Introduction

Loquat (*Eriobotrya japonica* Lindl) is a member of the Rosaceae family and is native to the southeast of China [1]. Carotenoids are the principal pigments in loquat and their concentration has been shown to be significantly correlated with fruit coloration [2,3,4]. At present, few studies of the regulatory mechanism of loquat carotenoids have been published due to the lack of information about its genome. With the development of modern molecular biological techniques, an investigation of the genetic mechanisms that regulate the accumulation of carotenoids in the loquat is timely.

Carotenoids are a major class of natural pigments of which more than 800 have been discovered [5,6]. Plant carotenoids are C_40_ isoprenoids with polyene chains that may contain up to 15 conjugated double bonds. They are important components of photosynthetic organisms due to their chemical properties [7]. They participate in the assembly of photosystems and perform a significant role in light capture, whereby they absorb light in a range of the blue spectrum broader than that achieved in chlorophyll. The excited carotenoids undergo excitation energy transfer to chlorophyll [8,9]. Carotenoids protect the photosynthetic apparatus from photooxidative damage caused by excess light energy through the quenching of both singlet and triplet state chlorophylls [9,10]. Carotenoids are conducive to produce scents and flavors that attract insects and animals for pollination and seed dispersal [11]. They are also significant precursors for the biosynthesis of metabolites, such as vitamin A, strigolactone, and abscisic acid [12,13,14,15,16]. Furthermore, carotenoids are thought to protect the human body from oxidative stress by removing free radicals, which can prevent cancer, cardiovascular diseases, and other chronic diseases [17,18,19].

Sites for the biosynthesis and accumulation of plant carotenoids are plastids, containing chloroplasts and chromoplasts [20]. The plant carotenoid biosynthesis and cleavage pathway is shown in Supplementary Figure S1. Geranylgeranyl diphosphate (GGPP), a precursor of carotenoid metabolism, is catalyzed to produce phytoene by phytoene synthase (PSY). Phytoene is the first carotenoid molecule and undergoes desaturation and isomerization to form lycopene. This process is catalyzed by desaturases (phytoene desaturase, PDS; ζ-carotene desaturase, ZDS) and isomerases (carotene isomerase, CRTISO; 15-cis-ζ-carotene isomerase, ZISO). Cyclization of lycopene is the key to the plant carotenoid biosynthetic pathway, consisting of ε-and β-cyclization. ε-cyclization reactions of lycopene eventually form lutein with the involvement of lycopene ε-cyclase (LCYE), lycopene β-cyclase (LCYB), β-carotene hydroxylase (BCH), and ε-carotene hydroxylase (ECH). β-cyclization reactions of lycopene undergo a series of reactions to form carotenoids, including β-carotene, β-cryptoxanthin, zeaxanthin, antheraxanthin, violaxanthin, and neoxanthin. In this process, multiple enzymes are involved in the synthesis of carotenoids, including LCYB, chromoplast-specific lycopene β-cyclase (CYC-B), BCH, zeaxanthin epoxidase (ZEP), violaxanthin de-epoxidase (VDE), and neoxanthin synthase (NXY) [9,10,21]. In pepper plants, capsanthin-capsorubin synthase (CCS) can catalyze the conversion of antheraxanthin/violaxanthin to capsanthin/capsorubin [7,9]. Carotenoid cleavage dioxygenases (CCD) and nine-cis-epoxycarotenoid dioxygenase (NCED) are involved in the carotenoid cleavage pathway in plants. Carotenoids can be cleaved and further modified to form strigolactone [15] and abscisic acid [13].

In the branch of β-cyclization, beta dominant mutations of the *CYC*-*B* gene encode a novel chromoplast-specific lycopene β-cyclase, which can catalyze cyclization of lycopene to form β-carotene and dramatically promote *CYC*-*B* expression levels in flowers and fruits at the breaker stage [22]. Studies have confirmed that the *CYC*-*B* gene is closely related to fruit coloration in the papaya and tomato [23,24]. The *CYC*-*B* gene serves as a signal for β-cyclization during the fruit ripening process and can boost β-carotene accumulation [25,26]. Nevertheless, a null allele of *CYC*-*B* results in the downregulation of β-carotene and the upregulation of lycopene. In addition, *CCS* also has the function of β-cyclase and plays a vital role in lycopene cyclization. Its amino acid sequence has close homology with CYC-B. Similarities in function, gene structure, and mapping position strongly suggest that the *CCS* and *CYC*-*B* genes are orthologs that have originated from a gene duplication event from a common ancestor, most likely *LCYB* [7]. So far, *CYC*-*B* gene has been widely studied in the tomato [23,27,28] and cloned from citrus [25], papaya [24], and other plants. However, few studies have explored the regulatory mechanisms of *CYC*-*B* gene in the loquat and its genetic mechanisms remain unclear.

Virus-induced gene silencing (VIGS) is an effective tool for analyzing plant gene function over a short period of time [29], as successfully utilized in the peach [30] and pear [31] from the Rosaceae family. As a technique for rapidly identifying gene function, VIGS is easy to perform. In this study, the *CYC*-*B* gene was silenced using a VIGS-mediated transient assay, in order to explore its regulatory mechanism in carotenoid accumulation. By revealing the function of the *CYC*-*B* gene, it is possible to obtain direct evidences for its involvement in carotenoid accumulation and coloration of loquat. And the use of the VIGS technique provides a new method for the research of loquat gene function.

## 2. Materials and Methods

### 2.1. Plant Materials

**Figure 1:**
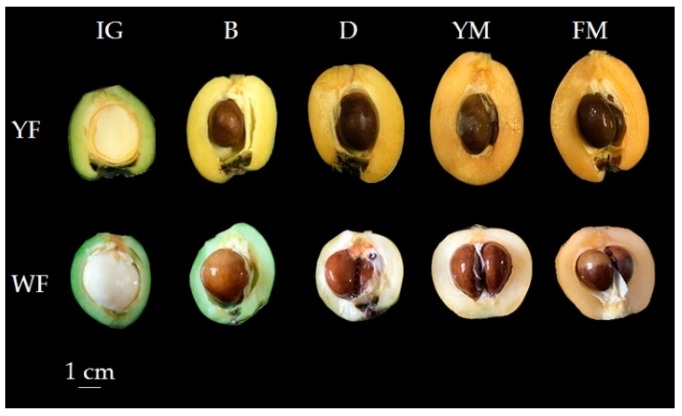
Color change of yellow-fleshed (YF) and white-fleshed (WF) loquats at different developmental stages. IG: immature green; B: breaker; D: degreening; YM: yellow mature; FM: fully mature.

Loquat fruits with different colors were sampled from the loquat germplasm resource preservation garden during 2016–2017 (College of Horticulture, Sichuan Agricultural University, Chengdu, China). ‘Dawuxing’ (yellow-fleshed, YF) and ‘Chuannong 1-5-9′ (white-fleshed, WF) fruits of different developmental stages were harvested for experiments, including immature green (IG), breaker (B), degreening (D), yellow mature (YM), and fully mature (FM) (Figure 1). ‘Zaozhong 6′ (yellow-fleshed) fruits at the breaker stage were selected as VIGS injection materials. The peel and flesh of the loquat fruits were frozen in liquid nitrogen, then immediately stored at −80 °C.

### 2.2. Identification of Loquat CYC-B Gene Full-Length cDNA and Phylogenetic Analysis

Loquat fruits were selected to extract total RNA, and first-strand cDNA was synthesized using a PrimeScript™ RT reagent Kit with gDNA Eraser (TaKaRa, Daliang, China), used as the template. A homology-based cloning method was selected because genome information of the loquat is incomplete. Degenerate primers containing the complete coding region (*cCYC-B*_F: 5′-CCACTTYCCTTTGACCTTCA-3′ and *cCYC-B*_R: 5′-GTGTTRCATTATTATGCAGCT-3′) were designed by this laboratory. A high-fidelity enzyme was used to amplify the *CYC*-*B* gene from ‘Dawuxing’, with homology alignment verified after sequencing. A phylogenetic analysis of CYC-B proteins from different plants was constructed using MEGA software (v5.2, https://www.megasoftware.net/) with neighbor-joining method (testing of 1000 bootstrap replications).

### 2.3. Plasmid Construction

**Figure 2:**
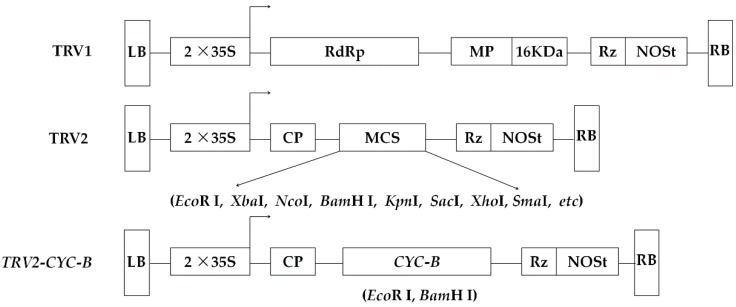
Construct maps of the TRV1, TRV2, and *TRV2-CYC-B* plasmids. A 543-length fragment of *CYC*-*B* gene was inserted into the multi-cloning site (MCS) of TRV2 plasmid. LB, left border; RB, right border; 2 × 35S, duplicated cauliflower mosaic virus (CaMV) 35S promoter; NOSt, nopaline synthase terminator; RdRp, RNA-dependent RNA polymerase; MP, movement protein; 16 KDa, cysteine enriched protein; CP, coat protein; Rz, self-cleaving ribozyme.

Construct maps of the TRV1, TRV2, and *TRV2-CYC-B* plasmids were shown in Figure 2. Primers with *Eco*R I and *Bam*H I restriction enzyme cutting sites (underlined) were designed in the conserved region of *CYC*-*B* gene sequence (*pCYC-B*_F: 5′-CGGGATCCATGGAGTTTGGGTTGATGAA-3′ and *pCYC-B*_R: 5′-CGGAATTCGCCTACTAACAAGCGAAGTT-3′). The fragment of *CYC*-*B* gene from ‘Zaozhong 6′ was inserted in an antisense orientation before the nopaline synthase (NOS) terminator and after the cauliflower mosaic virus (CaMV) 35S promoter. *CYC*-*B* fragment was purified using a SanPrep Column DNA gel extraction kit (Sangon, Shanghai, China). The purified product and TRV2 plasmid were both digested by *Eco*R I and *Bam*H I restriction enzymes. After ligating with T4 ligase overnight, it was transferred into DH5α *Escherichia coli* (*E. coli*) competent cells. *E*. *coli* was spread using selective media containing kanamycin (Kan, 50 mg/L). After expansion, the quality of *E. coli* was ascertained by running PCR and agarose gel electrophoresis. A pair of detection primer for *TRV2-CYC-B* (S1_F: 5′-CGGACGAGTGGACTTAGATTCTG-3′ and S1_R: 5′-GCCTATGGCTTCTGTTCATGTG-3′) were synthesized. *TRV2-CYC-B* plasmid DNA was extracted using a SanPrep Column Plasmid Mini-Preps kit (Sangon, Shanghai, China), as identified by *Eco*R I and *Bam*H I digestion then verified by sequencing.

### 2.4. Agrobacterium Transformation and Injection

*TRV2-CYC-B* and TRV1 and TRV2 plasmids were transfected into *Agrobacterium tumefaciens GV3101* competent cells by freeze-thaw. The method of Chen et al. [32] and Zhai et al. [31] were used for VIGS. *Agrobacterium* cells were spread in selective Luria Bertani (LB) solid medium containing Gentamicin (Gen, 40 mg/L), Kan (50 mg/L), and Rifampicin (Rif, 20 mg/L), then cultivated at 28 °C for 48 h–72 h. The growth of the colonies was observed. Recombinant, TRV1, and TRV2 colonies were inoculated with 50 mL of LB liquid medium containing 40 mg/L Gen, 50 mg/L Kan, 20 mg/L Rif, 10 mmol/L 2-morpholinoethanesulfonic acid (MES), and 20 μmol/L acetosyringone (AS), shaken at 28 °C until the OD 600 had risen to 1.2. The presence of the inserts was verified using 1 μL of bacterial solution as a template for PCR. TRV1 detection primers were created as described by Chen et al. [32]. *TRV2-CYC-B* detection primers were the same as S1 primers. TRV2 detection primers were as follows: TRV2_F: 5′-GCCATTAGCGACATCTAAAT-3′ and TRV2_R: 5′-CTAAGTCCACTCGTCCGTAA-3′. The verified cells were collected by centrifugation at 4000 g for 15 min and resuspended in infection buffer (containing MES 10 mmol/L, MgCl_2_ 10 mmol/L, AS 200 μmol/L) twice until the final OD-600 was 1.2. Finally, resuspended cells were gently shaken at 28 °C for 4 h.

Disease-free fruits of uniform size and maturity at the breaker stage were selected as injection materials. In this study, recombinant *TRV2-CYC-B* and TRV2 were mixed with TRV1 bacterial solution at a ratio of 1:1, respectively, using the empty TRV2 plasmid as a negative control. In addition, groups in which sterile water was injected, or not, were used. Twelve fruits of ‘Zaozhong 6′ in each group were selected, using a single fruit as a repeat. A loquat fruit was injected with 300 μL of mixed bacterial solution using sterile syringes. After a week, the pulp of the injection area (no necrosis) was cut into small pieces and immediately frozen in liquid nitrogen, then stored at −80 °C. The primers for viral molecular detection (the TRV1 and TRV2 primers described above) were used to amplify samples, in order to verify whether the TRV virus had successfully invaded the loquat fruits.

### 2.5. Quantification of Carotenoid Content

Twelve fruits of two groups (negative control or *TRV2-CYCB* silenced) were selected to determine total carotenoid content, respectively. Total carotenoid content in loquat samples was conducted using acetone extraction. After extraction using 80% acetone for 24 h, the OD at 470 nm of the supernatant was measured, with 80% acetone used as the control. Every fruit sample was performed in three replicates.

### 2.6. Transcription Level Analysis

Real-time quantitative PCR (qRT-PCR) was performed to quantify gene expression levels in fruits of different developmental stages and the VIGS samples (negative control or *TRV2-CYCB* silenced). Appropriate gene-specific primers of carotenoid biosynthesis were designed by Fu et al. [4]. A reaction mixture of 20 μL was performed using a CFX96 Real-Time PCR Detection System (Bio-Rad, USA), which included 10 μL SYBR Green I mix (2×), 1 μL gene-specific forward and reverse primers (10 μmol/L), 1.5 μL cDNA template (5 ng/μL), and 6.5 μL sterile water. The cycling procedure was as follows: 95 °C for 30 s, 95 °C for 5 s, 58 °C for 30 s, for 40 cycles. Analysis of the melt curve was performed in accordance with the instructions of the SYBR^®^ *Premix Ex Taq*™ II (TaKaRa, Daliang, China) kit. The 2^−ΔΔ*c*t^ values were computed to quantify relative gene expression levels [33]. Every qRT-PCR was performed in three replicates.

### 2.7. Statistical Analysis

In this study, IBM SPSS statistical software (v20, https://www.ibm.com/legal/copytrade.shtml) was used to perform Duncan’s pairwise comparison with a significance level of 5% (One-way Analysis of Variance, ANOVA, *p* < 0.05) and Pearson bivariate correlation analysis.

## 3. Results

### 3.1. Isolation of Loquat CYC-B Gene

**Figure 3:**
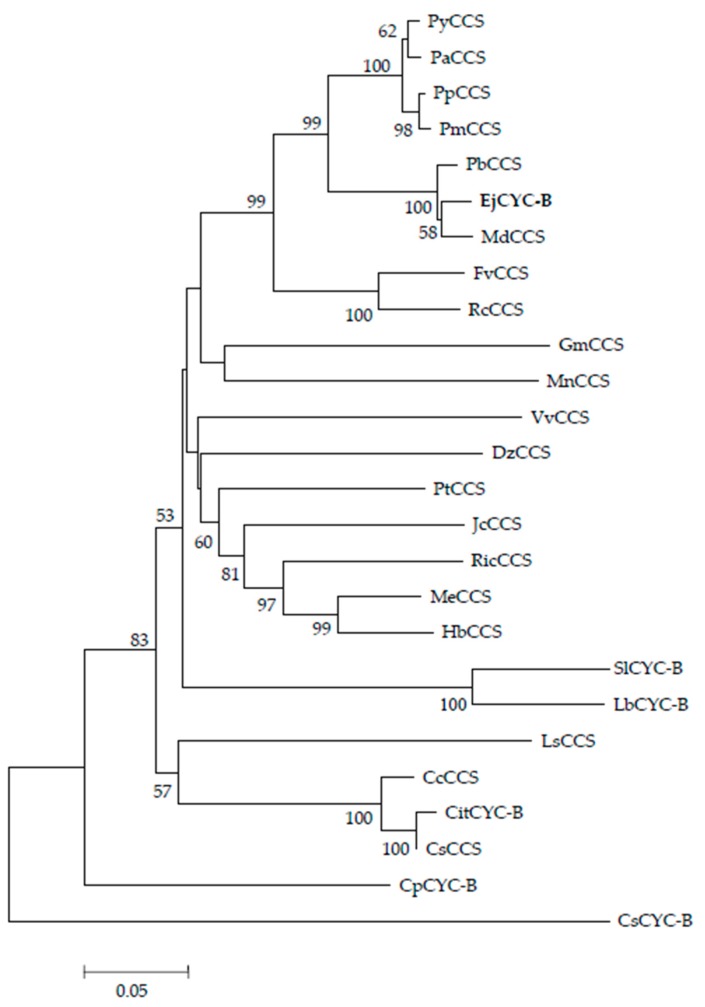
Phylogenetic tree of different plants. The results were obtained by neighbor-joining using MEGA software (v5.2) and the testing of 1000 bootstrap replications. GenBank Accession numbers were as follows: *Solanum lycopersicum* SlCYC-B AAG21133.1, *Citrus x paradisi* CitCYC-B ACX37456.1, *Crocus sativus* CsCYC-B ADA82241.1, *Lycium barbarum* LbCYC-B AIX87499.1, *Carica papaya* CpCYC-B APP91073.1, *Vitis vinifera* VvCCS NP_001304061.1, *Prunus yedoensis var. Nudiflora* PyCCS PQP92573.1, *Populus trichocarpa* PtCCS XP_002313611.2, *Ricinus communis* RicCCS XP_002523837.1, *Glycine max* GmCCS XP_003541965.1, *Fragaria vesca subsp. vesca* FvCCS XP_004291579.1, *Citrus clementina* CcCCS XP_006424195.1, *Citrus sinensis* CsCCS XP_006487912.1, *Prunus persica* PpCCS XP_007205102.1, *Prunus mume* PmCCS XP_008229097.1, *Malus domestica* MdCCS XP_008361015.2, *Pyrus x bretschneideri* PbCCS XP_009377819.1, *Morus notabilis* MnCCS XP_010102012.1, *Jatropha curcas* JcCCS XP_012070159.1, *Manihot esculenta* MeCCS XP_021627556.1, *Hevea brasiliensis* HbCCS XP_021678047.1, *Prunus avium* PaCCS XP_021805969.1, *Durio zibethinus* DzCCS XP_022753752.1, *Lactuca sativa* LsCCS XP_023756838.1, *Rosa chinensis* RcCCS XP_024156415.1.

In this study, the 1534 bp-length sequence of *CYC*-*B* gene was acquired containing full open reading frame and encoding a protein of 496 amino acids. The CYC-B protein sequence was used to perform a homology blast then conduct additional phylogenetic analysis (Figure 3). The loquat CYC-B protein had a phylogenetic relationship similar to other Rosaceae plants, of which the closest was *Pyrus x bretschneideri* and then was *Malus domestica*. The most distant genetic relationship was *Crocus sativus*. In addition, CYC-B and CCS proteins from *Citrus* were highly homologous. These results indicated that the isolated gene from the loquat belonged to the *chromoplast-specific lycopene β-cyclase* (*CYC*-*B*) gene. Full-length cDNA of the *CYC*-*B* gene was successfully isolated from the loquat, nominated as *EjCYC-B*.

### 3.2. Differential Accumulation Patterns of Yellow and White-Fleshed Loquats

**Figure 4:**
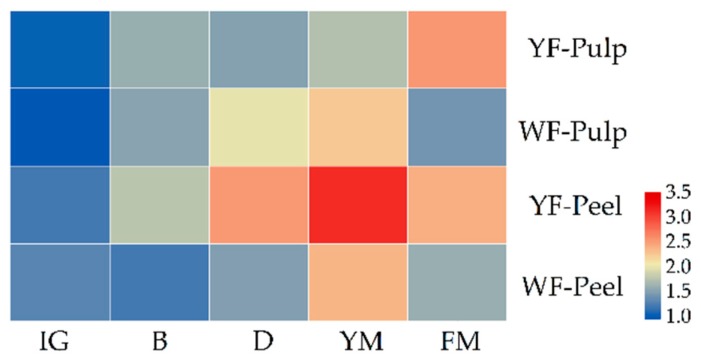
Relative *CYC*-*B* gene expression levels of yellow-fleshed (YF) and white-fleshed (WF) loquats in the pulp and peel at five developmental stages. The ‘Chuannong 1-5-9′ (WF) pulp at the IG stage was selected as the control to compute each 2^−ΔΔ*c*t^ value. The scale of intensity was represented as a log of base 2. Color intensity was presented as a legend.

The relative expression levels of the *CYC*-*B* gene demonstrated a rising trend before the YM stage, which was downregulated at the FM stage except for the pulp of ‘Dawuxing’ (Figure 4). Expression levels of the *CYC*-*B* gene were significantly different between loquats with different colors both in the pulp and peel after the B stage (*p* < 0.05). In the pulp, *CYC*-*B* gene expression of ‘Dawuxing’ and ‘Chuannong 1-5-9′ reached their maximum at the FM and YM stages, respectively. In the peel, *CYC*-*B* gene expression both reached its maximum at the YM stage. At the FM stage, the *CYC*-*B* gene expression level of ‘Dawuxing’ was approximately 3-fold higher than that of ‘Chuannong 1-5-9′ in the pulp, and approximately twice that of ‘Chuannong 1-5-9′ in the peel.

The total carotenoid content of loquats with different colors at different developmental stages was detected in our previous study (doi: 10.13417/j.gab.037.004407). The content of total carotenoids of ‘Dawuxing’ and ‘Chuannong 1-5-9′ varied greatly at different maturity stages. Pearson correlation analysis found that there was a significant and positive correlation between carotenoid content and *CYC*-*B* gene expression levels in the pulp of ‘Dawuxing’ at different developmental stages (*p* < 0.05). This result indicated that the change in *CYC*-*B* gene expression level may affect differential accumulation of carotenoids and coloration in the loquat.

### 3.3. Construction and Identification of Silencing Vector

After PCR amplification of the conserved region, a 543-length sequence was obtained. Comparison of homology with the *CCS* gene sequence of the pear (*Pyrus bretschneideri* Rehd.) (GenBank accession number: XM_009379544.2) demonstrated that sequence similarity reached 98.34%, indicating that the *CYC*-*B* gene fragment from loquat was successfully cloned and linked next to the TRV2 plasmid.

**Figure 5:**
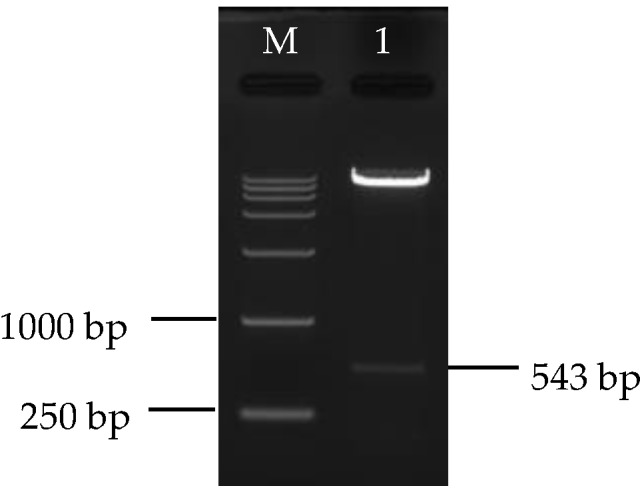
Enzyme digestion identification. 1 and M represented enzyme digestion products of *TRV2-CYC-B* and Marker III (TaKaRa, Daliang, China), respectively.

After double digestion of the recombinant plasmid, two distinct target bands appeared (Figure 5), and a target band corresponding to the length of the cloned fragment of the *CYC*-*B* gene obtained. The target gene region of the recombinant plasmid was sequenced, the result identical to that of the previous *CYC*-*B* gene cloned fragment. Therefore, one can conclude that the *TRV2-CYC-B* silencing vector had been successfully constructed.

### 3.4. TRV-Mediated CYC-B Gene Silencing Decreased Carotenoid Accumulation of the Loquat

**Figure 6:**
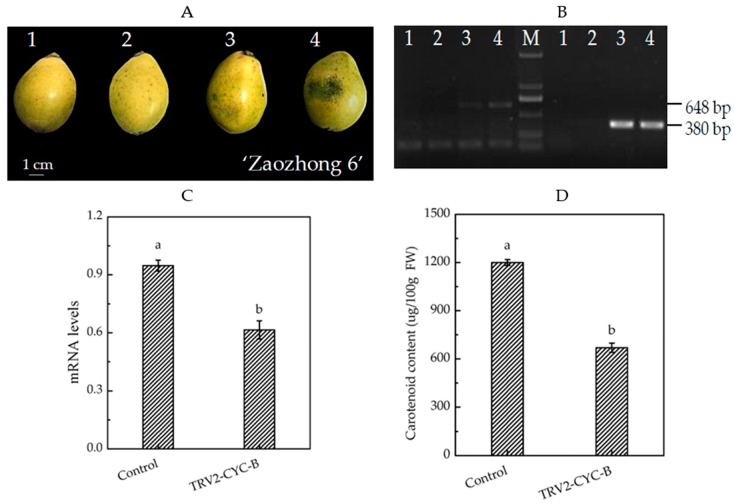
Carotenoid accumulation patterns in *VIGS-CYC-B* transient fruits. (**A**) Phenotypic comparison of loquat fruits that were not injected (1), injected with sterile water (2), negative control (3) and *TRV2-CYC-B* silenced (4). (**B**) PCR amplification of TRV1 (left) and TRV2 (right) in loquat pulp. The four templates were pulps of not injected (1), injected with sterile water (2), negative control (3) and *TRV2-CYC-B* silenced (4), respectively. M represented DL2000 Marker (TaKaRa, Daliang, China). qRT-PCR (**C**) and total carotenoid content (**D**) analysis in the pulp of negative control and silenced fruits. Different letters indicate significant differences between negative control and silenced fruits (One-way ANOVA, *p* < 0.05).

After infection by *Agrobacterium*, obvious phenotypic changes of the loquat fruits were observed. It was found that groups that were not injected or had been injected with sterile water and the negative control changed as normal from green to yellow, while the *TRV2-CYC-B* silenced fruits remained green (Figure 6A). Agarose gel electrophoresis detection found that TRV virus molecules were detected in the *TRV2-CYC-B* silenced group and negative control (Figure 6B). In addition, no TRV virus molecules were detected in groups not injected and those in which sterile water was injected. The results indicated that the TRV virus had successfully invaded the pulps of the loquat.

*CYC*-*B* gene expression in the pulp of *TRV2-CYC-B* silenced and negative control fruits was quantified by qRT-PCR. Compared with the negative control, *CYC*-*B* gene expression in the silenced fruits decreased significantly, to 64.8% of the negative control, silencing efficiency reaching 35.2% (Figure 6C). Total carotenoid content in the pulp of silenced fruits was 55.9% of the negative control, reduced by 44.1% (Figure 6D). Pearson correlation analysis found that expression levels of the *CYC*-*B* gene were significantly and positively correlated with total carotenoid content (*p* < 0.01). The results demonstrated that TRV-mediated *CYC*-*B* gene silencing resulted in a significant decrease in carotenoid accumulation in loquat pulp and interfered loquat coloration. The results also indicated that the VIGS system had been successfully constructed in the loquat.

### 3.5. Expression Analysis of Carotenoid Metabolism Related Genes after TRV-CYC-B Silencing

**Figure 7:**
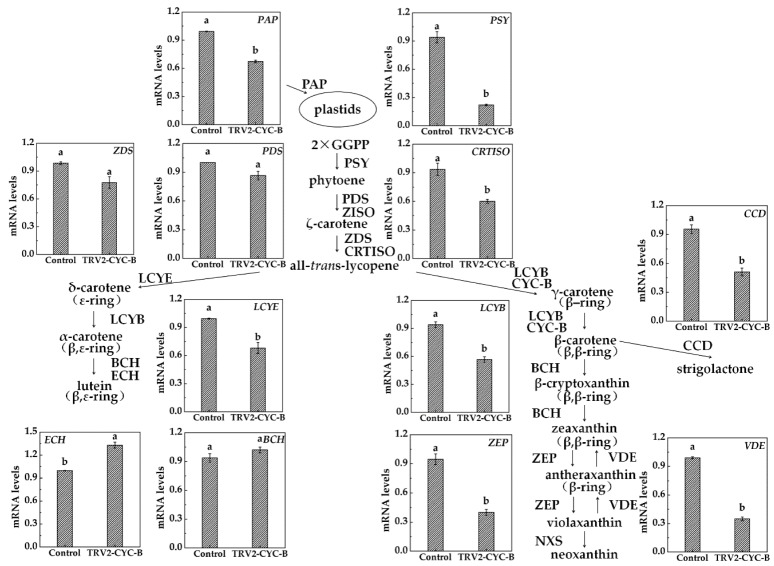
qRT-PCR analysis of genes related to carotenoid metabolic pathways after silencing. Plastids are the sites for the biosynthesis and accumulation of plant carotenoids. PAP, plastid lipid-associated protein; PSY, phytoene synthase; PDS, phytoene desaturase; ZDS, ζ-carotene desaturase; ZISO, 15-cis-ζ-carotene isomerase; CRTISO, carotene isomerase; LCYE, lycopene ε-cyclase; LCYB, lycopene β-cyclase; CYC-B, chromoplast-specific lycopene β-cyclase; BCH, β-carotene hydroxylase; ECH, ε-carotene hydroxylase; ZEP, zeaxanthin epoxidase; VDE, violaxanthin de-epoxidase; NXY, neoxanthin synthase; CCD, carotenoid cleavage dioxygenases. Different letters indicate significant differences between negative control and silenced fruits (One-way ANOVA, *p* < 0.05).

mRNA levels of 12 genes connected to carotenoid metabolism were analyzed in the pulp of *TRV2-CYC-B* silenced and negative control fruits (Figure 7). In linear carotenoid synthesis, there were no significant changes in the expression of *PDS* and *ZDS* genes, while the expression levels of *PSY* and *CRTISO* genes decreased significantly. In carotenoid cyclization branches, expression levels of *LCYB*, *LCYE*, *ZEP*, *VDE*, and *CCD* genes decreased significantly, while expression of the *ECH* gene increased significantly and that of the *BCH* gene did not change significantly. Additionally, *PAP* (plastid lipid-associated protein) mRNA levels also decreased significantly. To summarize, the expression of multiple structural genes involved in carotenoid accumulation changed after silencing the *CYC*-*B* gene.

## 4. Discussion

Carotenoids are the principal pigments in loquat, regulated by multiple factors. Since the discovery of the *chromoplast-specific lycopene β-cyclase* (*CYC*-*B*) gene by Ronen et al. [22], studies of the *CYC*-*B* gene become more widespread, finding that over-expression of *CYC*-*B* promoted formation of β-carotene in the tomato. Therefore, it is clear that the *CYC*-*B* gene plays an important role in the regulation of the accumulation of carotenoids. In this study, a *CYC*-*B* gene orthologue was successfully identified from the loquat using a homology-based cloning method, a simple, fast, and efficient method that uses highly conserved sequences to design degenerate primers to clone target genes. This has been used for carrots [34], *Phalaenopsis Hybrida* [35], and tobacco [36], etc.

In this study, the transcription levels of the *CYC*-*B* gene exhibited significant upregulation at the majority of developmental stages of yellow and white-fleshed loquats, as previously reported by Hadjipieri et al. [37]. At the FM stage, the expression of *CYC*-*B* gene was downregulated except in the pulp of ‘Dawuxing’. However, total carotenoid content increased at the FM stage in our previous study. We speculate that in addition to the *CYC*-*B* gene, other genes cooperate to regulate total carotenoid biosynthesis at the FM stage. Previous research has found that *PSY, CYCB,* and *BCH* genes have a synergistic effect on carotenoid accumulation [38]. In our previous study, the content of total carotenoids of ‘Dawuxing’ and ‘Chuannong 1-5-9′ varied greatly, first decreasing and then increasing. During loquat fruit maturation, the chloroplasts disintegrated and began to form chromoplasts, resulting in that chloroplast carotenoids decreased and chromoplast carotenoids increased. Fu et al. [4] have verified this through observation of the structure of loquat plastids. After the D stage, loquat fruits began to accumulate a large quantity of chromoplast carotenoids except for the pulp of ‘Chuannong 1-5-9′. In the pulp, carotenoid content of ‘Dawuxing’ was approximately 6-fold higher than that of ‘Chuannong 1-5-9′ at the FM stage. This result indicates that the pulp of ‘chuannong 1-5-9′ could not accumulate carotenoids effectively because of the failure to develop normal chromoplasts, exhibiting a white color, causing the difference in loquat pulp coloration. Fu et al. [4] also found the mature flesh of ‘Luoyangqing’ cultivars appeared red-orange due to the accumulation of abundant carotenoids and far fewer carotenoids accumulated in the flesh of white-fleshed cultivars.

After injection with *Agrobacterium*, the in vitro loquat fruits changed color from the breaker stage, fruit phenotype demonstrating obvious changes. It was found that there was shrinkage in the infected region of the silenced fruits, which we speculate is caused by the pressure difference of the infected region caused by enlarged interstitial spaces after excessive bacterial liquid injection. Following *CYC*-*B* gene silencing, the infected part of the silenced fruits retained a green phenotype, suggesting a delay in fruit maturity and interfering loquat coloration. In this study, decreased expression levels of the *CYC*-*B* gene were found in the pulp, and as a result of VIGS, led to additional reduction in carotenoid content, indicating that the *CYC*-*B* gene could positively regulate carotenoid accumulation in the loquat. So, we further confirmed that the *CYC*-*B* gene was closely related to loquat coloration, as found in other studies [23,24]. In addition, we found *CYC*-*B* gene silencing efficiency was 35.2%, while total carotenoid content reduced by 44.1%, indicating that the *CYC*-*B* gene played a key role in the regulation of carotenoid synthesis. Furthermore, this laboratory constructed the *TRV-PSY* silencing vector and found the silencing efficiency was 55.6%, total carotenoid content reducing by 46.8%. Additional experiments were conducted to explore why a lower silencing efficiency of *CYC*-*B* gene also caused a large drop in carotenoid concentration. After interfering with *CYC*-*B* expression, apart from the *PDS*, *ZDS*, and *BCH* genes, eight genes in the carotenoid metabolism pathway synergistically responded to downregulation of *CYC*-*B* gene expression, and the expression levels of seven genes (*PSY*, *CRTISO*, *LCYB*, *LCYE*, *ZEP*, *VDE*, *CCD*) were downregulated, except for the *ECH* gene. We speculate that suppression of multiple key structural genes may lead to a large reduction in carotenoids. Although *ECH* gene expression levels were upregulated, metabolized substrate synthesis of *ECH* gene was downregulated, which would reduce the synthesis and accumulation of carotenoids from the source. Moreover, downregulation of *PAP* gene expression might affect the development of plasmid structure and disturb the accumulation of carotenoids. Leitner-Dagan et al. [39] also found that inhibition of *CHRC* (a cucumber *PAP*) expression by RNAi resulted in a 30% decrease in carotenoid content in tomato flowers.

In this study, we found that the downregulation of the *CYC*-*B* gene after silencing was not complete. These factors influencing this are likely to be a selection of silencing vector [40], temperature [41,42], length and location of inserted fragments, and the similarity with target gene sequences [43]. VIGS was first successfully applied to *Nicotiana benthamiana*, with a modified tobacco mosaic virus (TMV) vector used to silence the *PDS* gene of *N. benthamiana* [44]. A variety of VIGS vectors have been developed based on a TMV vector for different hosts. The virus vectors suitable for different host plants affect systematic silencing in plants, thus resulting in different silencing efficiency. Previous research has found that temperature plays a key role in a plant’s silencing phenotype [41,42]. Studies have found that a lower temperature is conducive to maintenance of gene silencing for longer periods of time. In addition, a high similarity between inserted fragments and target gene sequences, with an inserted fragment of 200–1300 bp, located in the center of the cDNA sequence, and avoiding the silencing ends of the sequence also promote good silencing efficiency when using VIGS [43]. VIGS is an approach for the transient induction of RNA silencing in plants and has a subsequent symptom recovery stage. Thus, timely harvesting after infection is conducive to detection of high silencing efficiency. In this study, poor silencing efficiency may be due to inappropriate harvesting time.

The biosynthesis of plant carotenoids is a complex process, regulated not only by structural genes [9]. Moreover, cauliflower *OR* transgene regulates the accumulation of carotenoids by controlling differentiation and formation of chromoplasts [45]. Zhou et al. [46] discovered that the *OR* gene regulates carotenoid biosynthesis by controlling *PSY* gene expression, revealing a novel regulatory mechanism of carotenoids. Phytochrome interacting factors (PIFs) are a central factor in light-mediated reactions [47], which regulate *PSY* gene expression and carotenoid accumulation by binding to G-Box element on *PSY* gene promoter, and transducing light signals [48]. Environmental factors and chemical substances also affect carotenoid biosynthesis. While different factors have differential effects on carotenoid biosynthesis, they are possibly jointly involved in the carotenoid metabolic process, whose regulation mechanism requires additional elucidation. At present, genomic information of the loquat is incomplete. VIGS, a technique for rapid identification of gene function, is helpful for exploration of metabolic mechanisms of loquat.

## 5. Conclusions

TRV-mediated VIGS was used to cause effective silencing of the *CYC*-*B* gene, finding that the *CYC*-*B* gene positively regulated carotenoid accumulation and affects coloration in the loquat. Moreover, there was a multiple-gene synergistic response to the downregulation of *CYC*-*B* gene expression in the carotenoid metabolic pathway. These results directly indicate that the *CYC*-*B* gene plays a vital role in carotenoid accumulation and coloration in the loquat. Clarifying *CYC*-*B* gene function is of great significance for breeding new loquat varieties.

## Supplementary Materials

The following are available online at https://www.mdpi.com/2218-273X/9/12/874/s1, the sequence of the cloned *CYC*-*B* gene, Figure S1: The carotenoid biosynthesis and cleavage pathway in plants (modified from Moise et al. [9] and Colasuonno et al. [21]), Figure S2: The three-dimensional structure of *CYC*-*B* protein.
