# Supplementary material for: Expression of a Chromoplast-Specific Lycopene β-Cyclase Gene (CYC-B) Is Implicated in Carotenoid Accumulation and Coloration in the Loquat
Source: Biomolecules. 2019 Dec 13;9(12):874. doi: 10.3390/biom9120874 (PMC6995616; doi:10.3390/biom9120874)
Supplement: Supplementary file 1 [file biomolecules-09-00874-s001.pdf]

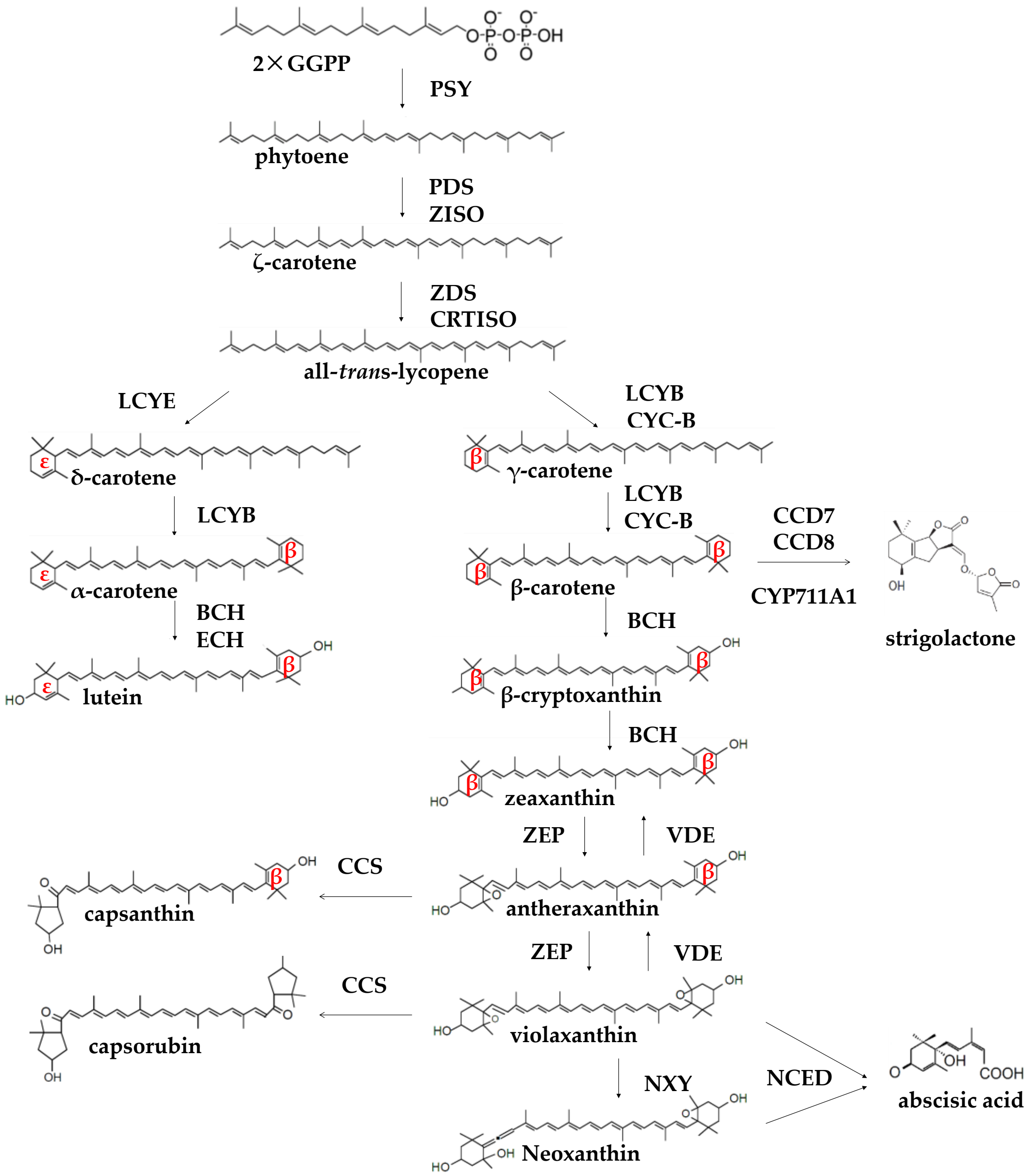

Figure S1. The carotenoid biosynthesis and cleavage pathway in plants (modified from Moise et al. [9] and Colasuonno et al. [21]). PSY, phytoene synthase; PDS, phytoene desaturase; ZDS, ζ-carotene desaturase; ZISO, 15-cis-ζ-carotene isomerase; CRTISO, carotene isomerase; LCYE, lycopene ε-cyclase; LCYB, lycopene β-cyclase; CYCB, chromoplast-specific lycopene β-cyclase; BCH, β-carotene hydroxylase; ECH, ε-carotene hydroxylase; ZEP, zeaxanthin epoxidase; VDE, violaxanthin de-epoxidase; NXY, neoxanthin synthase; CCS, capsanthin-capsorubin synthase; CCD7, (9′,10′) carotenoid cleavage dioxygenases; CCD8, (13,14) carotenoid cleavage dioxygenases; CYP711A1, cytochrome P450-type enzyme; NCED, nine-cis-epoxycarotenoid dioxygenase.

The sequence of the cloned *CYC-B* gene:

CCACTTTCCTTTGACCTTCATTCCTCGCTCTAATGGCCACCCTGCTCCGGCCATTTCCACCGCCAC
CATCCGCCGCCAAAACTTCCCAATTCTTCCACTCCTCCGGTCCTCCTCTCCATTTCTCCAAAACC
CATTACCCATCTCCAAATAAATCTCTCTCCAAAATCCACAGCAGCAAGTTCGGCAACTTTCTCG
ACTTAAAACCAGAGGCAAAACCCGAGTATTTGCACTTCGATCTCCATCAATTCGACCCGTCAAC
CCGGTCTCGCTTAGATGTGATCATCATCGGGACCGGCCCGGCCGGGCTTCGCCTTGCGGAGCAA
CTTTCTCGCTACGGCATTAAGGTATGCTGCGTTGATCCTTCTCCTCTTTCTATGTGGCCAAGTAAC

TATGGAGTTTGGGTTGAAGAATTTGAAAGCTTGAATCTTGAAAGTTGCTTGGACAAAATATGGC
CTATGGCTTCTGTTCATGTGAATGATAGTAAGACTAAGTTTTTGGACCGCCCTTATGGCAGAGTC
AGTAGGAAGAAACTCAAGACTTTGTTGCTGGAGAGGTGTCTCTCGAATGGGGTTCAATTTCATA
GGGCCAAGGTTTGGAAAATCGAACACGAAGAGTTCGAGTCTTCGATTTTGTGTGATGATGGGAA
TGAGCTCAAGGCAAGCTTGATTGTTGATGCTAGTGGGTTTGCAAGCAGTTTCGTAGAGTATGAG
AAGCCTAGGAACCATGGATATCAGATTGCTCATGGTATCTTGGCTGAAGTGGAAGAACACCCCT
TTGATTTGGATAAGATGCTTCTGATGGATTGGAGAGATTCCCATCTCGGAAACGAGCCTTATTTG
CGCACTAGTAATTCTAGATTTCCAACTTTTTTGTATGCAATGCCGTTTGATTCGAACTTGGTGTTT
TTGGAAGAAACTTCGCTTGTTAGTAGGCCGGTGTTGTCTTATATGGAGATTAAGAAACGAATGG
TTGCAAGGCTAAGGCATTTGGGGATTAGAGTGAAGAGGGTAATTGAAGAGGAGAAGTGTTTGA
TCCCAATGGGGGGTCCGCTTCCTCGGATCCCCCAACGTGTGATGGCAATTGGAGGGACTTCTGG
GGTGGTTCACCCTTCGACTGGGTACATGGTGGCTCGGACCATGGCTCTAGCCCCAGTATTGGCT
GAAGCCATTGCAGAGTGCCTTGGCTCAACCAGAATGATCCGAGGGCAGCCGCTTTATCATAGA
GCGTGGAATGGCTTGTGGCCAATTGAGAGGAGATGCACGAGGGACTTTTACTCATTTGGTATGG
AGACTTTGTTGAAGCTTGATCTGAATGGGAGTAGAAGCTTCTTTGACGCTTTCTTTGACTTGGAT
CCCTATTACTGGCAAGGCTTTTTATCGTCAAGGCTGTCTCTAAGAGAGCTTGCTTTGTTGAGCTT
ATCTCTGTTTGGCCGAGCCTCCGCCCCATCTAGGTTTGATATGGTTACAAAGTGTCCTGTGCCCT
TGGTTAAACTGATGGGCAATCTCGCACTTGAAGCTGCATAATAATGTAACAC

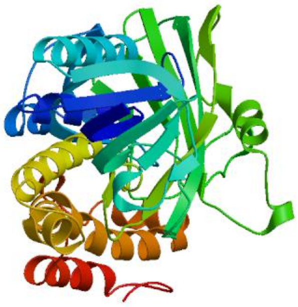

Figure S2. The three dimensional structure prediction of CYCB protein
